# Variance-based sensitivity analysis of tuberculosis transmission models

**DOI:** 10.1098/rsif.2022.0413

**Published:** 2022-11-23

**Authors:** Tom Sumner, Richard G. White

**Affiliations:** TB Modelling Group, TB Centre, Centre for Mathematical Modelling of Infectious Diseases, Department of Infectious Disease Epidemiology, London School of Hygiene and Tropical Medicine, London WC1E 7HT, UK

**Keywords:** tuberculosis, sensitivity analysis, modelling

## Abstract

Mathematical models are widely used to provide evidence to inform policies for tuberculosis (TB) control. These models contain many sources of input uncertainty including the choice of model structure, parameter values and input data. Quantifying the role of these different sources of input uncertainty on the model outputs is important for understanding model dynamics and improving evidence for policy making. In this paper, we applied the Sobol sensitivity analysis method to a TB transmission model used to simulate the effects of a hypothetical population-wide screening strategy. We demonstrated how the method can be used to quantify the importance of both model parameters and model structure and how the analysis can be conducted on groups of inputs. Uncertainty in the model outputs was dominated by uncertainty in the intervention parameters. The important inputs were context dependent, depending on the setting, time horizon and outcome measure considered. In particular, the choice of model structure had an increasing effect on output uncertainty in high TB incidence settings. Grouping inputs identified the same influential inputs. Wider use of the Sobol method could inform ongoing development of infectious disease models and improve the use of modelling evidence in decision making.

## 1. Introduction

Mathematical models are widely used to simulate the epidemiology of tuberculosis (TB) and provide evidence to inform policies to reduce TB burden. These models contain many sources of input uncertainty including the choice of model structure, parameter values and input data.

Previous analyses have shown that all these different sources of uncertainty can be important in determining the model outputs [1–3]. Despite this, many TB modelling studies focus on parameter uncertainty [4,5], and uncertainty in model structure and other inputs is either not considered or explored using a scenario-based approach. Such scenarios are often selected on an ad hoc basis, based on prior beliefs about the likely importance of different inputs. This approach does not allow for a quantitative comparison of the effect of different sources of input uncertainty and may miss potential interactions between uncertain inputs.

Systematic sensitivity analysis (SA) of how model inputs drive model outputs can be a critical part of the modelling process [6]. SA can help to understand model dynamics, identify opportunities for model simplification and pinpoint where additional data collection, to reduce uncertainty in model inputs, may improve the precision of evidence used for decision making. Methods for conducting SA can be broadly divided into local and global approaches. In local methods, inputs are varied one at a time, usually by some fixed fraction around their nominal value. Local methods do not explore the full input space or account for interactions between inputs. By contrast, global methods explore the full range of input uncertainties, typically by sampling from prior probability density functions, and account for potential interactions between inputs by varying all inputs simultaneously. A variety of global SA methods exist. Among the most commonly used in TB modelling are partial rank correlation coefficients (PRCCs) e.g. [4,7–9]. One limitation of PRCCs is that they assume a monotonic relationship between the model inputs and model outputs [6,10]. If this assumption is not satisfied, the PRCCs may provide incorrect results. Methods which do not have this limitation will be more generally applicable to infectious disease modelling and avoid the need to check assumptions of monotonicity prior to conducting an analysis. Previous analysis of TB models has found a non-monotonic relationship between the incidence of disease and the effect of preventive treatment [11] suggesting the use of alternative methods to study TB models is merited.

The Sobol method [12] is a global SA method that, unlike PRCCs, is model independent: it does not depend on any assumptions about the input–output relationship. The Sobol method can also provide information on the individual effects of model inputs on the model outputs and the additional effect due to interactions between inputs. This information is not provided by the calculation of PRCCs. The Sobol method is widely used in other modelling disciplines but has not been widely adopted in TB modelling or in infectious disease modelling more broadly [13], with few examples of its application found in the literature [14–16]. In this paper, we apply the Sobol method to a TB transmission model used to simulate the effects of a population-wide screening strategy. We demonstrate the use of the method to quantify the importance of both model parameters and model structure and show how it can be used to analyse the importance of groups of inputs to increase computational efficiency.

## 2. Methods

The first section of the methods describes the Sobol sensitivity analysis method (further details can be found in the electronic supplementary material, file S1) and the approach used to analyse groups of inputs. The second section describes the TB model used to demonstrate the method. All analysis was conducted using the R programming language and all code is available at https://github.com/tomsumner/Variance_SA_TB.

### 2.1. Sobol method

The Sobol method is a quantitative global SA method. Sensitivity of the model output to uncertainty in the model inputs is quantified in terms of the contribution of the inputs (either individually or in interactions with other inputs) to the total variance in the model outputs. For a model of the form

Y=f(X),

where $X=(X_{1},\ldots X_{k})$ is a vector of *k* uncertain inputs, the total variance in the output, *Y*, can be written as follows:

$$V(Y)=\overset{k}{\underset{i=1}{\sum }}V_{i}+\underset{1\leq i<j\leq k}{\sum }V_{ij}+\ldots +V_{1,2,\ldots ,k},$$

where *V_i_* is the contribution to the total variance due to input *i* and *V_i__j_* is the contribution due to the interaction between inputs *i* and *j* and so on. The sensitivity indices are obtained by normalizing the partial variances by the total variance. For example, the first-order sensitivity indices *S_i_*, which describe the reduction in the model output variance that could be obtained by fixing *X_i_*, are given by

$$S_{i}=\frac{V_{i}}{V(Y)}.$$

Second-order and higher order indices can be calculated to describe the effects of interactions; however, the calculation of all 2*^k^* − 1 indices required to completely characterize the input–output relationship is impractical for models with a large number of inputs.

As an alternative to calculating all higher order indices, the total sensitivity indices, *T_i_*, describe the total effect of input i on the model output, accounting for all possible interactions with other inputs. The total indices are given by

$$T_{i}=1-\frac{V_{∼i}}{V(Y)},$$

where *V*_∼*i*_ is the variance due to all other inputs except *i*. If there are no interactions between inputs, then the *S_i_* and *T_i_* are equal and the sum of all *S_i_* = 1. If *T_i_* > *S_i_* this indicates that the effect of input *i* is in part due to its interaction with other inputs.

The *S_i_* and *T_i_* can be calculated using Monte Carlo integrals based on sampling the distributions of the *k* model inputs. Following the method outlined by Homma & Saltelli [17], this requires the generation of two *N* by *k* matrices (where *N* is the number of samples drawn and *k* is the number of uncertain inputs). The model is evaluated for each set of inputs in these matrices as well as for combinations of the two matrices (further details are given in the electronic supplementary material, file S1). As a result, the set of first-order and total sensitivity indices can be calculated at a cost of *N*(*k* + 2) model evaluations. We used the R sensobol package [18] to generate the input samples and calculate the sensitivity indices. This implementation uses Sobol sequences to generate the input samples. These are quasi-random low-discrepancy sequences which provide better coverage than randomly generated numbers in high dimensions. In addition, the sample size can be continually increased without affecting the randomization. This is a useful property for testing convergence of the indices without having to pre-specify the maximum sample size.

### 2.2. Grouping inputs

As the number of model runs required for the Sobol method depends on the number of inputs *k*, its application to models with large numbers of inputs or long run times can be computationally intensive. In addition, it can be difficult to interpret the sensitivity indices for a large number of inputs.

An alternative approach is to group the *k* individual inputs into *g* < *k* classes [19,20]. For each class, *n* realizations of the inputs are generated by sampling from the input distributions of the members of that class. Each realization is assigned an integer number from 1 to *n* and the samples for calculating the Sobol indices of the classes are drawn from these discrete uniform distributions. The analysis of *g* groups requires *N*(*g* + 2) model evaluations.

### 2.3. Checking convergence, variability and importance

As the sensitivity indices are estimated using Monte Carlo samples to approximate integrals, they are subject to sampling variability. One result of this is that inputs which do not contribute to the output variance may be found to have non-zero sensitivity indices as a result of the numerical approximation.

We use non-parametric bootstrapping to calculate 95% confidence intervals for the sensitivity indices. Following Khorashadi Zadeh *et al*. [21], we then calculate the sensitivity indices for a ‘dummy’ input which has no influence on the model outputs. Model inputs whose sensitivity indices overlap with those of the dummy input can be considered non-influential. Using the method in [21] the ‘dummy’ indices can be calculated with no additional model evaluations.

We also check for convergence of the sensitivity indices by calculating their values for increasing values of *N* up to *N* = 5000. We compare the visual convergence of the indices using both the individual input and grouped input approaches.

### 2.4. Tuberculosis model

In this work, we use a simple compartmental TB model to illustrate the application of the SA method to different sources of input uncertainty and the grouping of inputs.

**Figure 1.**
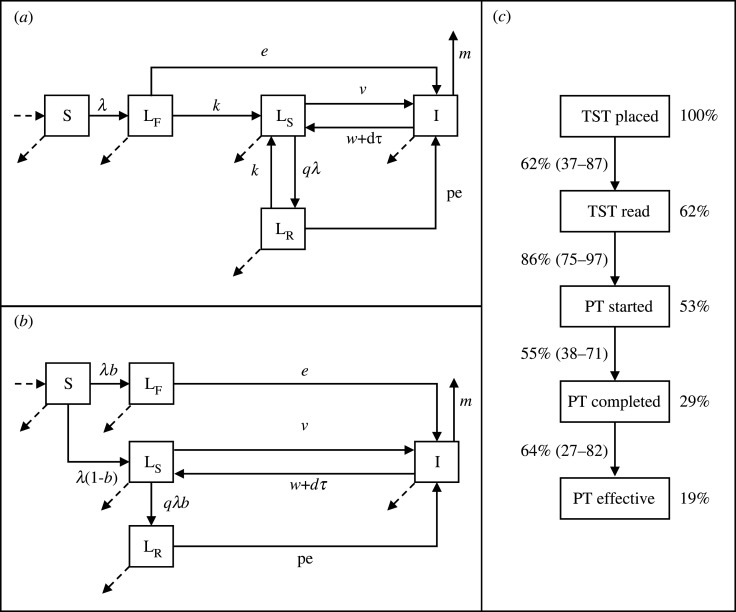
Model structure. (*a*) Core model structure with serial exposed states. (*b*) Core model structure with parallel exposed states. In both (*a*) and (*b*), dashed arrows represent births and natural mortality. (*c*) Preventive treatment care cascade. Values on the left-hand side indicate the proportion completing the step (median (95% range)); values on the right-hand side indicate the cumulative proportion retained at each stage (based on median proportions at each step). S = susceptible, L_F_ = recently exposed, L_S_ = remotely exposed, L_R_ = recently re-exposed, I = active TB, TST = tuberculin skin test, PT = preventive treatment. See table 1 for parameter definitions.

The model, illustrated in figure 1 (see electronic supplementary material, file S2 for model equations), follows the core structure used in many published TB modelling papers. Susceptible individuals (S) can be infected with *Mycobacterium tuberculosis* (M.tb) after which they enter a series of exposed (but non-infectious) states (L_F_ ,L_S_) from which they can progress to infectious TB disease (I). Individuals with TB disease experience an excess mortality risk (*m*) can naturally recover at a rate w or be diagnosed (at rate d). A proportion (*τ*) of diagnosed individuals is successfully treated and return to the L_S_ state, the remainder remain in the infectious disease state. Distributions for the core model parameters are taken from the literature and are shown in table 1.

**Table 1.** Input distributions. Distributions are fitted to 95% CIs taken from the literature using the rriskDistributions package (https://cran.r-project.org/web/packages/rriskDistributions/index.html). For the input data (TB incidence, CDR, treatment success (*τ*)) ranges reported by WHO are assumed to represent lower and upper bounds of uniform distributions. TB = tuberculosis, CDR = case detection ratio, ACF = active case finding, PT = preventive therapy, TST = tuberculin skin test.

input	description	median (95% CI)	distribution	source
parameters				
*m*	annual rate of death from TB	0.231 (0.112–0.350)	lognormal(−1.47,0.21)	calculated from estimates of pre-treatment disease duration and case fatality and prevalence of smear positivity [22–24]
*w*	annual rate of self-cure from TB	0.329 (0.208–0.450)	lognormal(−1.11,0.17)	
*k* (model 1)	annual rate of transition from recent infection to remote infection	4.015 (3.1025–5.11)	lognormal(1.39,0.13)	[25]
*b* (model 2)	proportion entering remote infection state following infection	0.91 (0.89–0.93)	beta(730.9,72.5)	
*e* (model 1)	annual rate of progression to TB disease from recent infection	0.4015 (0.3066–0.5475)	lognormal(−0.91,0.14)	
*e* (model 2)	annual rate of progression to TB disease from recent infection	4.015 (3.358–5.475)	lognormal(1.39,0.09)	
*V*	annual rate of progression to TB disease from remote infection	2.01 × 10^−3^ (9.13 × 10^−4^–4.02 × 10^−3^)	lognormal(−6.2,0.37)	
*q*/*p*	relative risk of infection (*q*) or disease (*p*) in previously infected individuals (versus naive)	0.59 (0.56–0.67)	beta(270.6,180.7)	[26]
model choice				
*M*	model structure used to represent progression from infection to disease (figure 1)	–	uniform(0,1)	–
	*M* >= 0.5: model 1			
	*M* < 0.5: model 2			
*R*	mechanism of protection due to prior infection (figure 1)	–	uniform(0,1)	–
	*R* >= 0.5: mechanism 1 (*q* < 1, *p* = 1)			
	*R* < 0.5: mechanism 2 (*q* = 1, *p* < 1)			
data				
TB incidence	baseline TB incidence per 100 000 population—global analysis	127 (114 140)	lognormal(4.8,0.05)	[27]
	baseline TB incidence per 100 000 population—high incidence (Philippines) analysis	539 (306–838)	lognormal(6.3,0.24)	
CDR	proportion of incident TB cases notified and started on treatment. Used to derive the diagnostic rate (d) in the model	–	uniform(0.53,0.66)	
*τ*	proportion of TB cases starting treatment who successfully complete treatment	–	uniform(0.8,0.9)	
ACF				
ACF sensitivity	the sensitivity for TB of the diagnostic algorithm used in the ACF intervention—assume universal Xpert	0.69 (0.48–0.86)	beta(14.6,6.7)	[28]
treatment uptake	the proportion of TB cases diagnosed via the ACF intervention who start TB treatment	0.77 (0.71–0.82)	beta(172.9,51.9)	
PT				
TST sensitivity	the sensitivity of TST for diagnosing M.tb infection	0.77 (0.71–0.82)	beta(172.9,51.9)	[29]
TST completion	the proportion of people having TST placed who return for test results	0.62 (0.37–0.87)	beta(8.4,5.2)	[30] values for general population cohorts
PT uptake	the proportion of people testing positive via TST who start PT	0.86 (0.75–0.97)	beta(43.7,7.3)	
PT completion	the proportion of people starting PT who complete PT	0.55 (0.38–0.71)	beta(18.3,15.3)	
PT efficacy	the proportion of people completing PT who are protected against progression to TB disease	0.64 (0.27–0.82)	beta(7.8,13.6)	[31]

To explore the impact of the choice of model structure on model outputs, we include two different representations of the exposed states and progression to disease: (i) serial exposed states in which all infected individuals enter the L_F_ state and transition to the L_S_ state at rate *k* (figure 1*a*); (ii) parallel exposed states in which a proportion (*b*) of infected individuals enter the L_F_ state and the remainder enter the L_S_ state (figure 1*b*)). In both cases, L_F_ represents a recent exposure state with a high risk (*e*) of progression to disease and L_S_ a remote exposure state with a lower risk (*v*) of progression. Previous analysis [25,32] has shown that these two structures perform equally when replicating data on the incidence of TB by time since infection. As a result, we assume equal probability for each structure in the SA.

It is typically assumed that prior exposure to M.tb confers some degree of protection against subsequent infection and/or disease. To account for the uncertainty around the mechanism of this protection, we consider two different ways in which it is included in the model: (i) prior infection reduces the risk of re-infection (*q* < 1, *p* = 1 in figure 1); (ii) prior infection reduces the risk of progressing to disease if re-infected (*q* = 1, *p* < 1 in figure 1). As for the choice of progression model structure, we assume equal probability for each mechanism of protection in the SA.

### 2.5. Input data

To explore the role of uncertainty in input data, we used WHO estimates of global TB incidence, case detection rate (CDR, the proportion of incident cases that are notified) and treatment success (*τ*) as inputs to the model [27]. The CDR is used to determine the rate, *d*, at which individuals with TB disease are diagnosed and started on treatment. The distributions for these inputs are shown in table 1.

We fit the model to the WHO global incidence estimates by varying the contact parameter, *β*, which determines the rate of infection, *λ* = *β*I. This is done by solving the steady state solution of the model for *β* for each set of sampled inputs (model choice, parameters and input data). Details of the steady state solutions are given in the electronic supplementary material, file S2.

In a previous qualitative analysis of the effect of model structure on the predicted impact of TB preventive therapy (PT) [2], we found that the relative importance of the choice of model structure varied with the baseline TB incidence in the model. To explore how the results of the SA depend on the setting being modelled, we repeated the analysis using WHO estimates of TB incidence for the Philippines, an example of one of the 30 WHO classified high TB incidence countries, with an estimated TB incidence of 539/100 000 (306–838) [27].

### 2.6. Intervention

We model a hypothetical one-off screening of the population (for simplicity we model the screening as an instantaneous event). The intervention consists of both active case finding (ACF) for prevalent TB disease and PT for those with M.tb infection. We assume that everyone is tested for TB with an Xpert-like test with estimates of test sensitivity taken from WHO TB screening guidelines [28]. For simplicity, we ignore false positive diagnosis (i.e. we assume that the test has 100% specificity). Of those testing positive, a proportion (given by the ‘Treatment uptake’ parameter [28]) start treatment. Those starting treatments after ACF have the same probability of treatment success (*τ*) as those diagnosed in the baseline model.

We also assume everyone is screened for infection with M.tb with a tuberculin skin test (TST) and that those who test positive are offered PT. We use estimates of TST sensitivity from a previous systematic review [29] and as for TB testing ignore false positive diagnosis. We include several steps in the process from attending for an initial TST to completing preventive treatment using the findings of a systematic review of the cascade of care in the diagnosis and treatment of M.tb infection [30]. These are shown in figure 1*c*. Of those completing PT a proportion (given by the PT efficacy) move to a post-PT state where they have zero risk of progressing to TB disease but can be re-infected.

Distributions for all the intervention parameters are also shown in table 1.

### 2.7. Grouping inputs

The model has a total of *k* = 20 uncertain inputs. For the grouped SA, we assign these 20 inputs into five groups: ‘Model’ group (two inputs: the choice of structure for disease progression, the choice of mechanism of protection due to prior infection); ‘Parameters’ group (eight inputs: the parameters of the core TB model); ‘Data’ group (three inputs: baseline TB incidence, CDR and treatment success); ‘ACF’ group (two inputs: test sensitivity, treatment uptake); ‘PT’ group (five inputs: TST sensitivity, TST completion, PT uptake, PT completion, PT efficacy). For the ‘Model’ group, there are two options for each input, resulting in four possible realizations to sample. For each other group we generate *n* = 10 000 realizations for the group by sampling from the distributions in table 1.

## 3. Results

**Figure 2.**
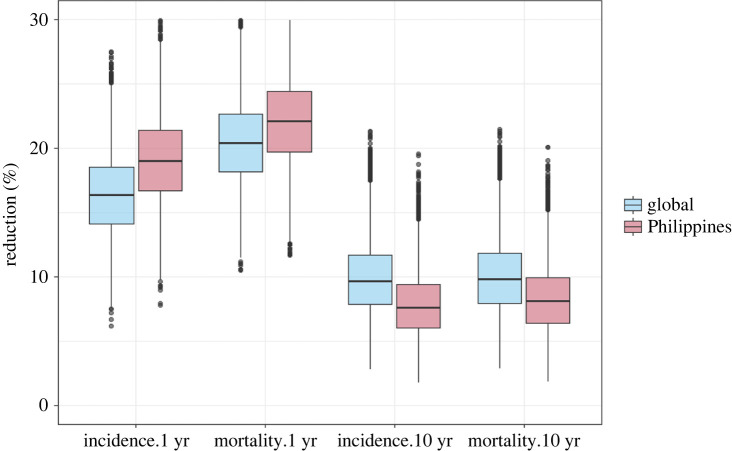
Model estimates of percentage reduction in TB incidence and mortality. Blue: global TB incidence setting; pink: high TB incidence (Philippines) setting. Boxes show the first and third quartiles (the 25th and 75th percentiles), horizontal lines the median. Whiskers extend to 1.5 times the interquartile range and points indicate outliers.

Figure 2 shows the range of the outputs of the model (percentage reductions in TB incidence and TB mortality over 1 and 10 years compared with baseline values) when all inputs are sampled from the distributions in table 1. The greatest reductions are predicted in mortality after 1 year, with smaller reductions in incidence over the same time period. For both measures, the reduction is lower over 10 years as the TB burden returns toward baseline values following the one-off screening intervention. The short-term impacts (1 year) are larger in the high TB incidence setting (Philippines (pink) versus global (blue)). In the longer term (10 years), the situation is reversed. The lower long-term effect in the high-incidence setting results from the increased risk of infection in this setting, which in turn results in a reduction in the long-term benefit of PT. This supports the view that repeated rounds of screening will be required to achieve sustained reductions in TB incidence, as observed in a recent trial of ACF in Vietnam [33].

**Figure 3.**
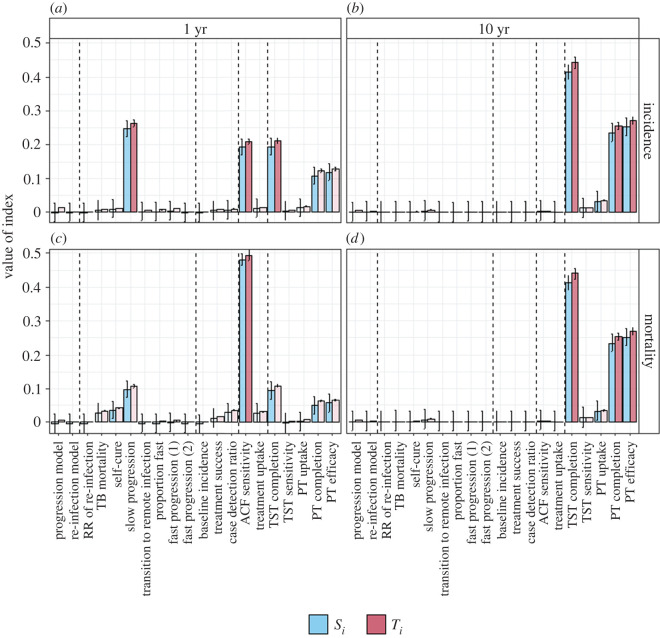
Results of individual Sobol analysis. Panels show results for different outputs. (*a*,*b*) Reduction in TB incidence; (*c*,*d*) reduction in TB mortality; (*a*,*c*) 1 year time horizon; (*b*,*d*) 10 year time horizon. Blue bars show the individual indices (*S_i_*), red bars the total effects (*T_i_*). Error bars show 95% confidence intervals based on bootstrapping. Lighter shading indicates inputs that are non-influential based on comparison with the dummy indices. Dashed vertical lines divide the individual inputs into the groups used in the grouped analysis.

The following describes the results of the SA applied to the global TB incidence setting. Figure 3 shows the sensitivity indices (main and total) for the individual model inputs. For the longer term impacts (10 years, right-hand column of figure 3) on both incidence and mortality, the model is most sensitive to the PT parameters. Approximately 40% of the variance is explained by the TST completion parameter and approximately 25% by each of PT completion and PT efficacy. For the short-term impacts (1 year), the slow-progression rate and the ACF sensitivity are identified as important inputs in addition to the PT parameters. For all inputs, the difference between the main and total effects is small, suggesting that interactions play a minor part in the output uncertainty. This is consistent with the sum of the *S*_i_s which range from 0.89 to 0.96 across the four outputs considered, suggesting approximately 90% of the variance in the output is explained by individual inputs.

**Figure 4.**
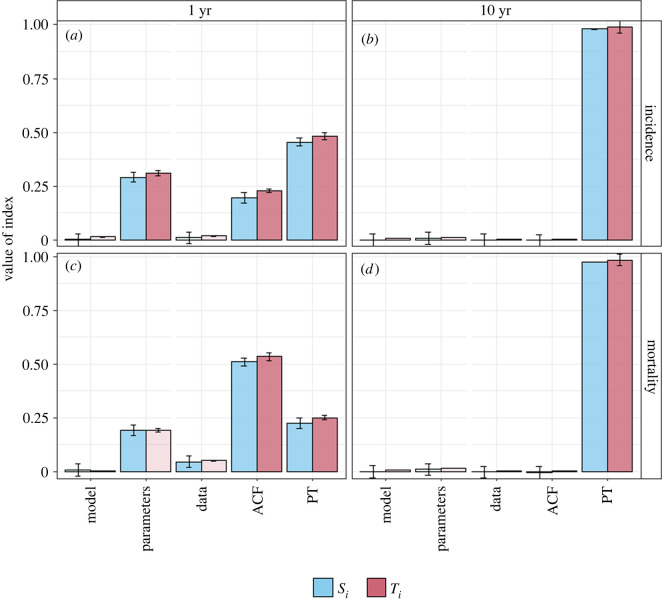
Results of grouped Sobol analysis. Panels show results for different outputs. (*a*,*b*) Reduction in TB incidence; (*c*,*d*) reduction in TB mortality; (*a*,*c*) 1 year time horizon; (*b*,*d*) 10 year time horizon. Blue bars show the individual indices (*S_i_*), red bars the total effects (*T_i_*). Error bars show 95% confidence intervals based on bootstrapping. Lighter shading indicates inputs that are non-influential based on comparison with the dummy indices.

Figure 4 shows the results of the grouped analysis. Consistent with the individual analysis, we find that the ‘PT’ group is responsible for almost all the variance in the predicted reduction in incidence and mortality over 10 years.

For the reductions over 1 year, the ‘Parameters’ group (cf. slow progression in the individual analysis) and ‘ACF’ group (cf. ACF sensitivity in the individual analysis) are also found to be influential. In each case, the group indices are approximately equal to the sum of the individual indices within each group.

For both the individual and grouped analysis, we found that the estimated indices were stable with sample sizes of *N* = 2500 or greater (see electronic supplementary material, file S3, figures A1 and A2). For the individual analysis with *k* = 20, this corresponds to 55 000 model evaluations. By contrast, the grouped analysis (*g* = 5) requires 17 500 model evaluations.

**Figure 5.**
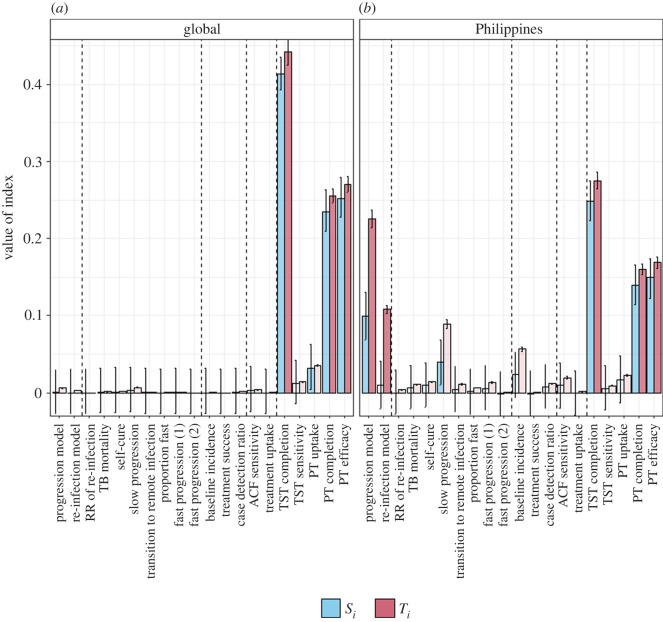
Comparison of sensitivity indices for different incidence settings. (*a*) Global TB incidence; (*b*) example of high TB incidence country. Blue bars show the individual indices (*S_i_*), pink bars the total effects (*T_i_*). Error bars show 95% confidence intervals based on bootstrapping. Lighter shading indicates inputs that are non-influential based on comparison with the dummy indices.

Figure 5 compares the sensitivity indices for the reduction in incidence over 10 years using the WHO estimates of global TB incidence (figure 5*a*) and the example of a high TB incidence setting (the Philippines, figure 5*b*). In a high-incidence setting, the choice of progression model is identified as an additional important input based on its main effect (accounting for approximately 10% of the variance). In the high-incidence setting, there is also an increased role of interactions between inputs, as shown by the larger difference between the main and total effects. In particular, the choice of re-infection model is important due to its interactions. The sum of the main effect indices (*S*_i_) is 0.77 (compared with 0.95 in the global incidence setting) indicating that more than 20% of the variance in the output is due to interactions between the uncertain inputs. Full results for the high TB incidence setting are shown in the electronic supplementary material, file S3 (electronic supplementary material, figures A3 and A4).

## 4. Discussion

The results of our analysis showed that uncertainty in the intervention parameters was more important than uncertainty in the core model inputs in determining the variance in the predicted long-term reductions in TB incidence and mortality. This finding is supported by a recent model analysis of a mass TB screening intervention [34] which found that the model outcomes (TB cases and deaths averted) were robust to the core model parameters.

We also found that the inputs identified as important are context specific, with the most important inputs depending on the choice of output, time horizon and the setting considered. In particular, we found that the model structure used to represent disease progression and the mechanism of protection provided by prior infection were important inputs when a high TB incidence setting was modelled. This is consistent with our previous qualitative analysis of disease progression assumptions in TB modelling in which we found that the choice of model structure was increasingly important at higher TB incidence [2].

The results of the Sobol analysis provided useful information for future development of the model. The results suggested that improved estimates of the PT parameters would have the biggest effect in improving the precision of model predictions. Conversely, many of the core model inputs could be fixed without loss of information.

Grouping inputs were found to provide results consistent with the individual analysis, identifying the groups that contained the influential individual inputs as important. The use of groups also reduced the computation time of the analysis. In our example application, we found that the sample size, *N*, required to obtain stable estimates of the Sobol indices was similar for both the individual and grouped analysis. As a result, the reduction in the number of model iterations required for the grouped analysis depended on the ratio of (*g* + 2) to (*k* + 2) (where *g* is the number of groups and *k* the number of individual inputs). In our example (*g* = 5 and *k* = 20), grouping inputs resulted in an approximate two-thirds reduction in the number of model iterations. Analysing groups of inputs could also be used as a preliminary screening step to reduce the number of inputs to be included in an individual analysis.

The Sobol method required distributions to be specified for each input. The results of the analysis may be dependent on these distributions. In our analysis, we defined input distributions based on published estimates for the model inputs where possible. However, using other literature sources may have resulted in different input distributions and changes in the Sobol indices. We do not consider this aspect in our analysis, but methods to assess the robustness of the Sobol indices to uncertainty in the input distributions have been proposed elsewhere [35,36].

While uncertainty and SA is often incorporated into infectious disease modelling studies, wider use of the Sobol method could inform ongoing development of models and improve the use of modelling evidence in decision making.

## Data Availability

All code used in the analysis is available from: https://github.com/tomsumner/Variance_SA_TB. All model code used in this manuscript is available to download from: https://github.com/tomsumner/Variance_SA_TB. The data are provided in the electronic supplementary material [37].
